# Outcomes of thoracic endovascular aortic repair for complicated type B acute aortic dissection from a multicenter Japanese post-market surveillance study

**DOI:** 10.1007/s11748-025-02123-4

**Published:** 2025-02-01

**Authors:** Yoshimasa Seike, Sophie B. Green, Keita Mori, Kimberly Reid, Hitoshi Matsuda, the Vascular Investigators, Hitoshi Matsuda, Naotaka Motoyoshi, Masaki Hata, Hiroyuki Kamiya, Ichiro Ideta, Joji Fukada, Genta Chikazawa, Hiroshi Ishitoya, Takafumi Masai, Tetsuo Sonomura, Kenjiro Kaneko, Yoshikatsu Saiki, Hirokazu Minamimura, Shoichi Takahashi, Masanao Toma, Masafumi Morita, Yutaka Makino, Kazuo Abe, Shinichi Iwakoshi, Toshihiro Funatsu, Keiji Iwata, Ryuta Kiuchi, Kei Kazuno, Yoshiharu Nishimura, Masao Yoshitatsu, Hisashi Satoh, Shinichiro Shimura, Tetsuya Horai

**Affiliations:** 1 Department of Cardiovascular Surgery, National Cerebral and Cardiovascular Center, 6-1 Kishibeshimmachi, Suita, Osaka 564-8565 Japan; 2 W.L. Gore & Associates, Flagstaff, Arizona USA

**Keywords:** Type B aortic dissection, Thoracic endovascular repair, Clinical outcomes, Healthcare surveillance

## Abstract

**Objectives:**

A primary goal of thoracic endovascular aortic repair (TEVAR) for type B acute aortic dissection (BAAD) is exclusion of the primary entry tear with a suitable stent graft (SG) to reestablish true lumen flow and promote aortic remodeling. This study aimed to determine the safety and efficacy of a conformable thoracic SG in a Japanese population with complicated BAAD.

**Methods:**

Between 2016 and 2017, 43 patients with complicated BAAD were enrolled in this prospective, nonrandomized, multicenter post-market surveillance study at 27 sites in Japan. All patients underwent TEVAR using the Gore TAG Conformable Thoracic Endoprosthesis (CTAG) (W.L. Gore and Associates, Flagstaff, AZ).

**Results:**

The most common TEVAR indication for complicated BAAD was malperfusion (41.9%; 24 out of 43) and aortic rupture was observed in 32.5% of patients (14 out of 43). All SG implants were successfully completed and there was no patient with surgical conversion. Thirty-day mortality was 7.0% (3 out of 43) and one patient (2.3%) experienced spinal cord ischemia during hospitalization. Entry tear exclusion was achieved in 91.3% of patients at 1 month, and 95.7% at 24 months. Through 24 months after TEVAR, no retrograde type A aortic dissection was observed and distal stent graft induced new entry was observed in two patients (4.7%).

**Conclusion:**

TEVAR utilizing the CTAG device for complicated BAAD in Japan demonstrated a low incidence of perioperative mortality and complications. Complications directly attributed to the SG including RTAD and dSINE were uncommon and the midterm outcomes were deemed satisfactory.

**Supplementary Information:**

The online version contains supplementary material available at 10.1007/s11748-025-02123-4.

## Introduction

Complicated type B acute aortic dissection (BAAD) occurs in about 25–30% of all type B dissection, and its management is still controversial [1]. Aortic rupture and malperfusion to major visceral branches, lower extremities, and spinal cord are absolute indications for entry closure using thoracic endovascular aortic repair (TEVAR), which is recommended as Class I in the guidelines [2]. According to the Japanese Association of Thoracic Surgery, in 2019 among cases treated for acute Type B dissection, TEVAR procedures accounted for 526 cases (74.8%) out of 703 cases treated for BAAD [3]. However, it should be noted that complications of TEVAR for BAAD are more frequent than those of TEVAR for chronic type B dissection, which have been reported to be about three times as common [4, 5].

Several TEVAR devices have been approved to treat aortic dissection in Japan and evidence on the utility of these devices in the treatment of complicated BAAD is imperative. Preventing measures of severe complications, including stroke, spinal cord ischemia (SCI), retrograde type A aortic dissection (RTAD), and distal stent graft induced new entry (dSINE), are critical and device characteristics that do not cause damage to the aortic wall for preventing embolization and dissection is mandatory.

The Gore TAG Conformable Thoracic Endoprosthesis (CTAG) (W.L. Gore and Associates, Flagstaff, AZ) has provided acceptable clinical and anatomic outcomes for complicated BAAD in 26 investigative sites in the United States (US) [6]. In addition, a multicenter trial demonstrated the safety and effectiveness of the CTAG for the treatment of descending thoracic aortic aneurysms during a 5-year follow-up [7]. However, the outcomes of CTAG use for complicated BAAD in Japan have not been clarified. Hence, this study aimed to investigate the use of the CTAG through 2 years in a Japanese post-market surveillance (PMS) registry in complicated BAAD and to validate the safety and effectiveness of CTAG in this population group.

## Materials and methods

### Ethics statement, study design, and patient cohort

This Japanese PMS study was regulated by the Japanese Ministry of Health, Labor and Welfare, and conducted in accordance with Good Post-marketing Study Practice. Per regulatory requirements, each institution’s ethical committee determined whether the need for informed consent was necessary, or if outcome data could be extracted while protecting patient’s rights without requiring individual patient consent.

Between January 2016 and December 2017 following commercial approval of the CTAG to treat complicated BAAD in Japan, 43 patients were enrolled in a prospective nonrandomized multicenter PMS study at 27 sites in Japan (Clinicaltrials.gov no. NCT05414318).

### Inclusion/exclusion criteria

Before enrollment in the PMS study, patients underwent a physical examination and pre-procedure imaging, including computer tomography (CT) of the chest abdomen and pelvis presented with complicated BAAD. To be included in this PMS study, patients had to meet the following criteria: treating physician determined that treatment with CTAG was appropriate, subject did not respond to medical therapy positively, time from onset to dissection symptoms was less than 14 days, adequate iliac or femoral access, aortic inner diameter of proximal landing zone in the range of 16–42 mm, ≥ 20 mm-long landing zone proximal to the primary entry tear; no dissection of proximal extent of the landing zone. No patients were excluded from the PMS study, because they met these criteria.

### Follow-up

Patients had planned follow-up visits, including physical examination and imaging at 1 and 6 months, then annually through 5 years after the index procedure. This work reports data through 24 months of follow-up. Additionally, all-cause mortality, major device events, and serious adverse events were recorded.

### Device specifications

The CTAG is a thoracic endoprosthesis (i.e., stent graft [SG]) comprised of an expanded polytetrafluoroethylene (ePTFE) tube reinforced with ePTFE/fluorinated ethylene propylene film that is supported by a self-expanding nitinol (composed by nickel titanium alloy) wire frame along its external surface [6]. The instructions for use are stated in the section of *Inclusion/ Exclusion criteria*, and it is suggested to consider coverage of ≥ 10 cm distal to the primary entry tear and ensure that the distal end of the device is positioned in a straight portion of the aorta. The recommended oversizing rate for device selection was in the range of 6 ~ 33%, measured in the internal diameter of the proximal landing zone without dissection. However, the final actual device selection was based on the judgment of each facility. In addition, the decision to use either true luminal or aortic diameter as the basis for sizing the distal landing zone with dissection was made at the discretion of the institution. Commercial approval for the use of CTAG in complicated BAAD in Japan was granted April 17, 2015, and reimbursement started on January 1, 2016.

### Primary and secondary endpoints

The primary safety endpoint was survival during the follow-up periods. Aortic-related mortality was defined as death that occurred within 30 days of index procedure or death due to aortic rupture during the follow-up. This outcome was adjudicated by the authors at the conclusion of the study. The secondary safety endpoints were adverse events including stroke, respiratory failure, cardiac infarction, renal failure, SCI, bowel ischemia, RTAD, and dSINE after TEVAR. RTAD was defined as new intimal involving the aortic arch, and dSINE was defined as a new intimal tear distal to the SG occurring; these outcomes were adjudicated by the authors at the conclusion of the study.

The primary effectiveness endpoint was the achievement of primary entry tear exclusion. The secondary effectiveness endpoints included the prevention of adverse aortic events, comprising aortic rupture, re-intervention, and changes in true and false lumen diameters.

### Statistical analysis

Data were collected via a web-based electronic report form (Electronic Data Capture) to ensure secure authentication of data. Data management was performed by SRD Co., a limited liability company, which served as a contract research organization.

The statistical analysis was conducted using SAS version 9.4 for Windows (SAS Institute Inc., Cary, NC) and completed by the Gore Clinical Research Department (W. L. Gore and Associates). All data were reviewed for inconsistencies and where missing values were identified; sites were queried for resolution. Categorical variables were expressed as the number of subjects and percentage (%) of the overall group. Continuous variables were expressed as mean [standard deviation (SD)], median, and range. All-cause mortality, aortic-related mortality, and TEVAR-related complication (including dSINE, RTAD, and stroke) rates through end of 24-month follow-up window (post-procedure day 911 or 30.1 months) were estimated using Kaplan–Meier (KM) analysis and presented with KM curves, accompanied by corresponding 95% confidence intervals (CIs).

## Results

### Patients

A total of 43 patients were enrolled in this study and all patients (23.3% females) were treated by TEVAR using CTAG for complicated BAAD. Preoperative patient characteristics are presented in Table 1. Refractory shock was observed in 3 patients (7.0%). The most common TEVAR indication for complicated BAAD in this cohort was malperfusion (41.9%; 24 out of 43) and aortic rupture was observed in 32.5% of patients (14 out of 43). The mean days from symptom onset to diagnosis was 0.9 ± 2.7 days. One patient on day 15 of onset was deemed eligible for enrollment for safety and efficacy evaluation, though 14 days and a few hours had passed since onset (Table 1).

**Table 1:** Baseline demographics and presenting dissection characteristics

Number of enrolled subjects	43
Female	10 (23.3%)
Age (years)	
Mean (std dev)	64.3 (14.3)
Median (range)	65.0 (40, 89)
Body mass index	
Mean (std dev)	23.6 (4.1)
Median (range)	22.9 (16, 33)
Comorbidity	
Hypertension	29 (67.4%)
Cerebrovascular disease	6 (14.0%)
Diabetes mellitus	5 (11.6%)
Pulmonary disease	4 (9.3%)
Renal failure	2 (4.7%)
Connective tissue disease	1 (2.3%)
Refractory shock	3 (7.0%)
Indication for TEVAR	
Malperfusion	18 (41.9%)
Rupture	14 (32.5%)
Impending rupture	6 (13.9%)
Re-dissection	3 (7.0%)
Therapy-refractory pain	2 (4.7%)
Details of complications	
Rupture^*^	13 (30.2%)
Leg ischemia	12 (27.9%)
Mesenteric ischemia	12 (27.9%)
Renal ischemia	12 (27.9%)
Hepatic/Splenic ischemia	4 (9.3%)
Spinal cord ischemia	1 (2.3%)
Arm ischemia	0 (0.0%)
Indication for TEVAR	
Malperfusion	18 (41.9%)
Days from symptom onset to diagnosis	
Number of subjects **	40
Mean (std dev)	0.9 (2.7)
Median (range)	0.0 (0, 15)
< 7 Days	38 (88.4%)
7–14 Days	1 (2.3%)
> 14 Days	1 (2.3%)

*TEVAR* Thoracic endovascular aortic repair

^*^Hemorrhage outside of aortic boundaries differentiated from reactive effusions

^**^No data were available for 3 patients

### Early outcomes

A total of 62 CTAG SG were deployed during the study period, with an average of 1.4 ± 0.50 devices deployed per patient. In Japan, insurance only reimburses 2 SG per procedure; 55.8% of patients (24 out of 43) received 1 SG and 44.2% of patients (19 out of 43) received 2 SG. In the 19 patients who received 2 SG, the same size of SG was used in 4 and different sizes were used in the other 15. Data on the individual SGs including size and length were not available. Proximal and distal landing zones are provided for each patient in Appendix Table S1. Proximal landing zones ranged from 0 to 4, while distal landing zones ranged from 3 to 12.

Concomitant procedures performed with index procedure in 23 patients included surgical bypass in 13 patients [12 left subclavian artery (LSA) revascularization and 1 femorofemoral bypass], aortic stenting in 8 patients (5 aortic bare stent, 2 thoracic cuff, and 1 abdominal cuff), peripheral stenting in 6 patients (3 superior mesenteric artery, 1 renal, 1 common iliac, 1 unknown), and other treatments reported in 7 patients (6 LSA embolization, 1 exploratory laparotomy). There were no conversions to open surgical repair. The 30-day mortality rate was 7.0% (3 out of 43). One patient died during the index procedure from intestinal complications and the other 2 patients died from multiple system organ failures. In terms of perioperative complications, 1 patient (2.3%) experienced paraplegia, 1 (2.3%) patient experienced an access-related complication, and 4 patients (9.3%) experienced bowel complications. No stroke was reported in any of the patients. No endoleaks were reported during the index procedure or post-procedure during the hospitalization (Table 2). The median hospitalization time was 27 days (range 7–78 days).

**Table 2:** Adverse events and endoleaks

	Procedure *n* = 43	Post-procedure *n* = 42	1 Month *n* = 40	6 Months *n* = 40	12 Months *n* = 38	24 Months *n* = 35	Total *n* = 43
Aortic rupture	0 (0.0%)	1 (2.4%)	0 (0.0%)	0 (0.0%)	0 (0.0%)	1 (2.9%)	2 (4.7%)
Retrograde type A aortic dissection	0 (0.0%)	0 (0.0%)	0 (0.0%)	0 (0.0%)	0 (0.0%)	0 (0.0%)	0 (0.0%)
Distal stent graft induced new entry	0 (0.0%)	0 (0.0%)	2 (5.0%)	0 (0.0%)	0 (0.0%)	0 (0.0%)	2 (4.7%)
False lumen enlargement ≥ 5 mm	0 (0.0%)	1 (2.4%)	0 (0.0%)	0 (0.0%)	0 (0.0%)	0 (0.0%)	1 (2.3%)
Pulmonary	0 (0.0%)	0 (0.0%)	0 (0.0%)	2 (5.0%)	0 (0.0%)	0 (0.0%)	2 (4.7%)
Cardiac	0 (0.0%)	0 (0.0%)	0 (0.0%)	0 (0.0%)	1 (2.6%)	1 (2.9%)	2 (4.7%)
Bowel	1 (2.3%)	0 (0.0%)	0 (0.0%)	1 (2.5%)	1 (2.6%)	1 (2.9%)	4 (9.3%)
Vascular	1 (2.3%)	1 (2.4%)	0 (0.0%)	0 (0.0%)	0 (0.0%)	0 (0.0%)	2 (4.7%)
Bleeding	1 (2.3%)	0 (0.0%)	0 (0.0%)	0 (0.0%)	0 (0.0%)	0 (0.0%)	1 (2.3%)
Neurologic	0 (0.0%)	1 (2.4%)	0 (0.0%)	0 (0.0%)	0 (0.0%)	0 (0.0%)	1 (2.3%)
Infection	0 (0.0%)	0 (0.0%)	0 (0.0%)	1 (2.5%)	1 (2.6%)	1 (2.9%)	3 (7.0%)
Unplanned branch vessel occlusion	1 (2.3%)	0 (0.0%)	0 (0.0%)	0 (0.0%)	0 (0.0%)	0 (0.0%)	1 (2.3%)
Other complications (not device-related complication)	0 (0.0%)	2 (4.8%)	0 (0.0%)	1 (2.5%)	0 (0.0%)	0 (0.0%)	3 (7.0%)
Endoleak	0 (0.0%)	0 (0.0%)	2 (5.0%)	0 (0.0%)	1 (2.6%)	0 (0.0%)	3 (7.0%)
Type Ia	0 (0.0%)	0 (0.0%)	1 (2.5%)	0 (0.0%)	0 (0.0%)	0 (0.0%)	1 (2.3%)
Type II	0 (0.0%)	0 (0.0%)	0 (0.0%)	0 (0.0%)	1 (2.6%)	0 (0.0%)	1 (2.3%)
Type III	0 (0.0%)	0 (0.0%)	0 (0.0%)	0 (0.0%)	0 (0.0%)	0 (0.0%)	0 (0.0%)
Other	0 (0.0%)	0 (0.0%)	1 (2.5%)	0 (0.0%)	0 (0.0%)	0 (0.0%)	1 (2.3%)

### Survival and late adverse events

**Fig. 1:**
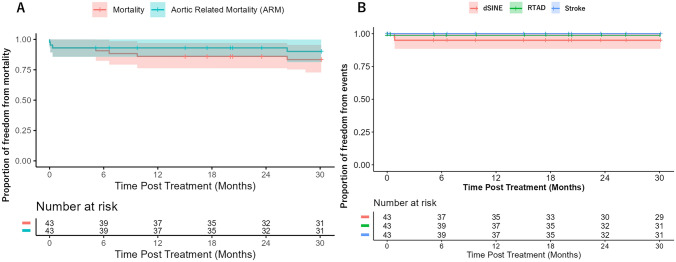
**A** Kaplan–Meier (KM) curves showing freedom from all-cause mortality and aortic-related mortality through 24 months of follow-up for 43 patients with acute complicated type B dissections post-thoracic endovascular aortic repair. KM analysis estimated freedom from all-cause mortality to be 83.4% (95% CI 68.2%, 91.7%), estimated freedom from aortic related to mortality to be 90.1% (95% CI 75.6%, 96.2%) through 24 months. **B** Kaplan–Meier (KM) curves showing freedom from distal stent-induced new entry (dSINE), retrograde type A dissections (RTAD), and strokes through 24 months of follow-up for 43 patients with acute complicated type B dissections post-thoracic endovascular aortic repair. KM analysis estimated freedom from RTAD and stroke to be 100%, estimated freedom from dSINE to be 95% (95% CI 81.5%, 98.7%) through 24 months

Through the 24-month window, 29 (82.9%) patients completed 24-month follow-up. Between 30 days and 24 months, 4 deaths were recorded. One patient died 797 days post-procedure due to an aortic rupture related to a graft infection in the ascending aorta, stemming from a previous surgery predating the TEVAR procedure. The other three patients died from causes unrelated to the device or TEVAR procedure: one patient died 155 days post-procedure from pneumonia, another patient died 200 days post-procedure due to laryngeal spasm during a tracheotomy tube replacement, and the third patient died 294 days post-procedure from sepsis. KM analysis estimated an 83.4% freedom from all -cause mortality (95% CI: 68.2%—91.7%) and 90.1% freedom from aortic-related mortality (CI 75.3%−96.2%) at 24 months (Fig. 1A).

During the 24-month follow-up, a total of 27 adverse events were documented and listed in Table 2. Among these, dSINE was observed in 2 patients and KM analysis estimated freedom from dSINE to be 95% (95% CI 81.5—98.7%). Through 24 months, there were no reported RTAD and stroke and KM estimated freedom from stroke and RTAD was 100% (Fig. 1B). Regarding other adverse events, bowel complications were observed in 3 patients through 24 months and one patient required antibiotic administration due to SG infection at 6-month mark (Table 2).

### Endoleaks and reinterventions during the follow-up

At 1 month after TEVAR, 2 patients (5.0%) exhibited a type Ia endoleak, and 1 (2.5%) presented with an indeterminable endoleak. At the 12-month mark following TEVAR, one patient (2.5%) presented type II endoleak originating from the intercostal artery (Table 2). During the follow-up period, 2 reinterventions were conducted for 2 patients. One patient underwent additional TEVAR using CTAG for aortic enlargement due to type Ia endoleak one month after initial TEVAR. The other patient underwent an additional CTAG placement with debranching bypass from the left carotid artery to the LSA for aortic enlargement due to a type Ia endoleak at the 24-month mark.

### Aortic changes and remodeling

**Fig. 2:**
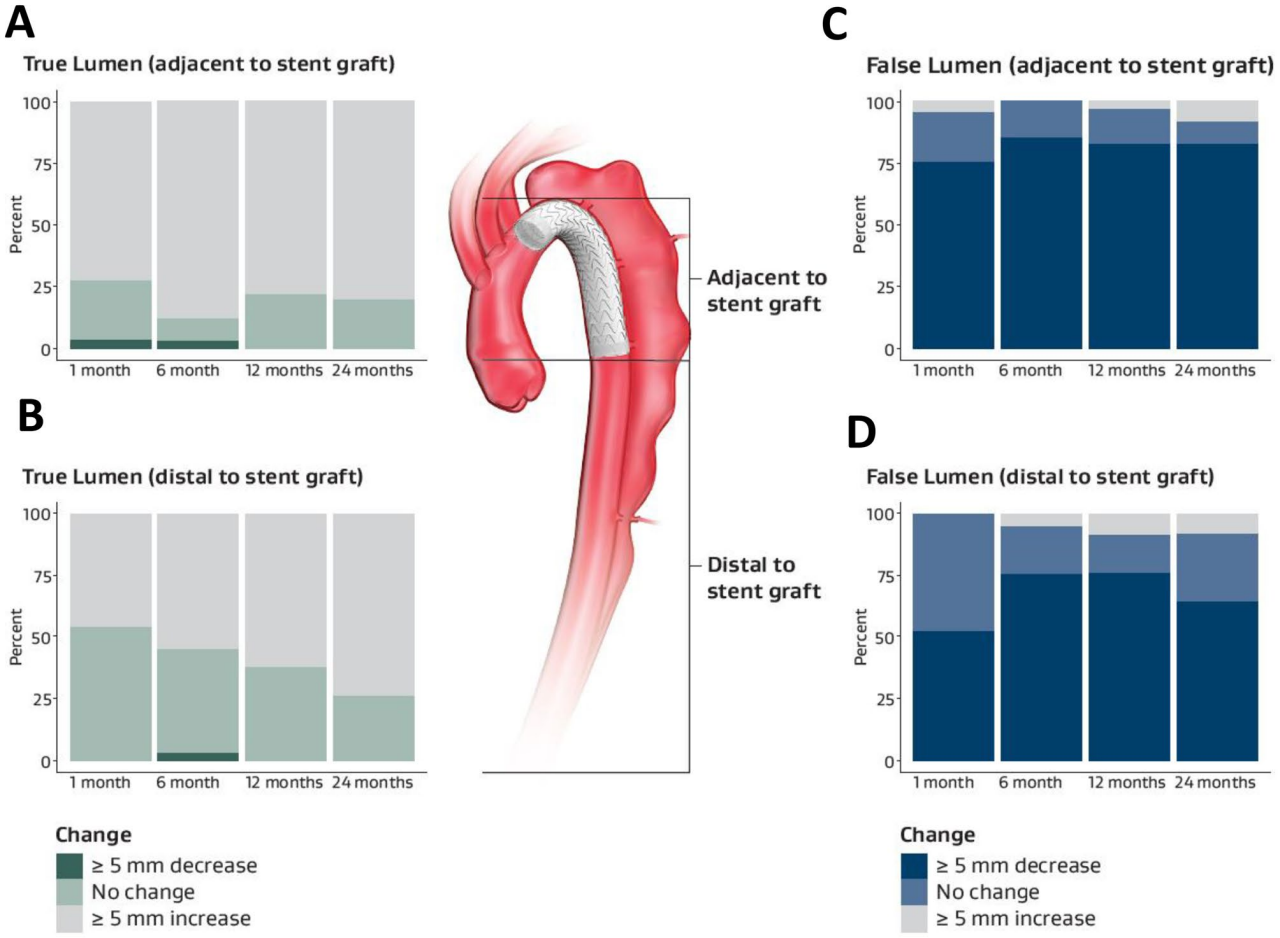
Changes in aortic remodeling of the false and true lumen at the site adjacent to the stent graft (SG) and distal, pre-procedure through 24 months: **A** true lumen adjacent to SG, **B** true lumen distal to SG, **C** false lumen adjacent to SG, and **D** false lumen distal to SG. Data are represented as % of the group having change > 5 mm, < 5 mm, or no change. True lumen data are minimum vales and false lumen are maximum values

**Fig. 3:**
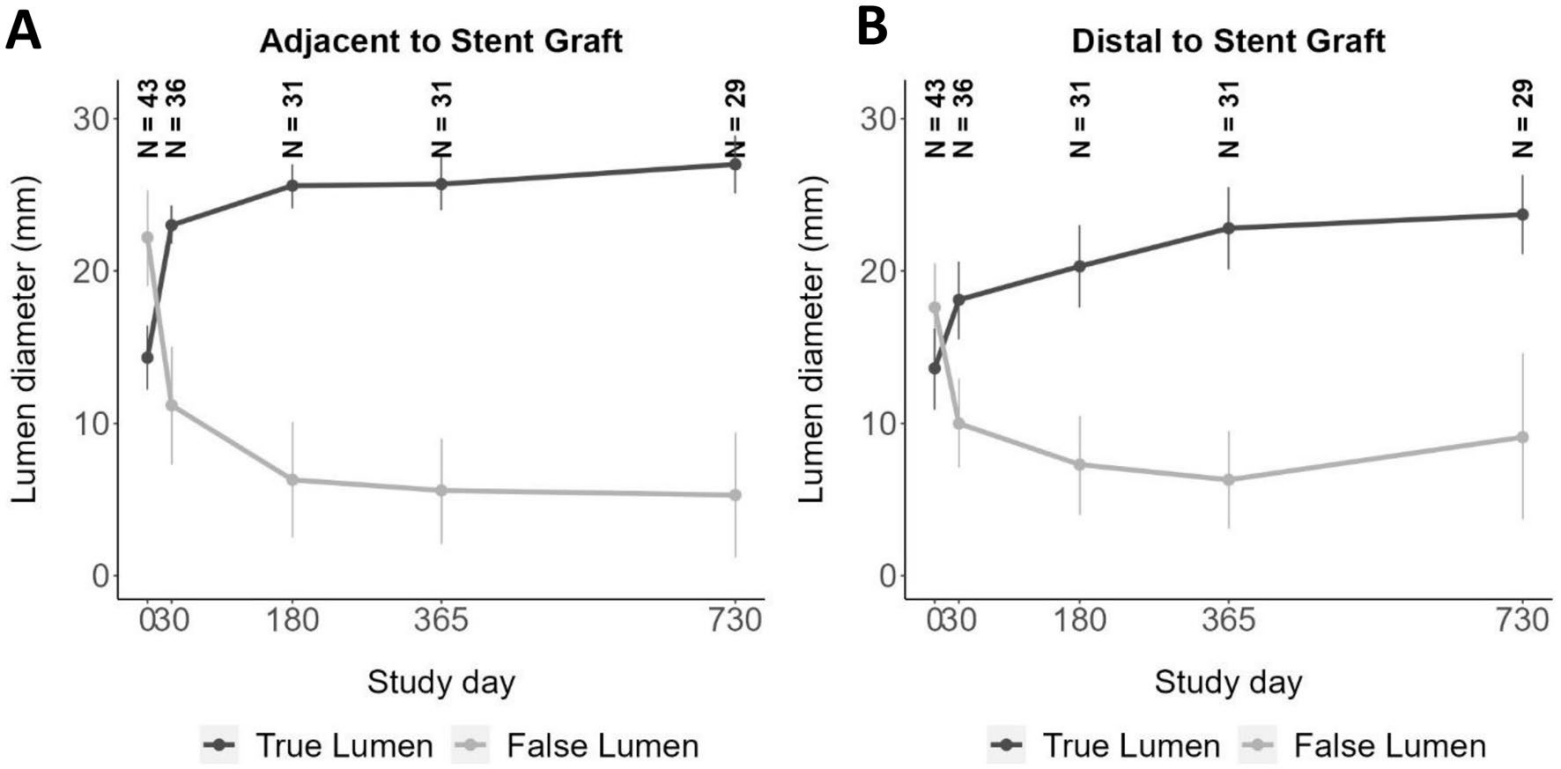
Changes in aortic remodeling of the false and true lumen at the site adjacent to the stent graft (SG) and distal, pre-procedure through 24 months: Average change in the true and false lumen over time (**A**) adjacent to SG and (**B**) distal to SG. Data are reported in mm, true lumen value are minimum vales, and false lumen are maximum values. Lumen diameter changes at both the adjacent segment and distal segment to SG exhibited an inverse relationship between the false and true lumen

Primary entry tear exclusion was observed in 91.3% (21 out of 23) of patients at 1-month mark on CT. During the follow-up, all patients had primary entry tear exclusions at 6- and 12-month follow and 95.7% (22 out of 23) patients at 24-month follow-up after initial TEVAR. Changes in aortic remodeling of the false and true lumen at the site adjacent to the SG and distal from pre-procedure to 24 months after TEVAR are shown in Fig. 2. At the 24-month mark, the true lumen had enlarged more than 5 mm and the false lumen had shrunk more than 5 mm at the site adjacent to SG in more than 75% of patients. At the site distal to SG, the true lumen was enlarged by more than 5 mm in more than 75% of patients and the false lumen was shrunk by more than 5 mm in more than half of the patients (Fig. 2). Lumen diameter changes at both the adjacent segment and distal segment to SG exhibited an inverse relationship between the false and true lumen (Fig. 3).

## Discussion

This study offers valuable information concerning the practical application of the CTAG device for treating complicated BAAD derived from a multicenter, prospective, PMS investigation within an exclusively Japanese population. In this study, patients with complicated BAAD underwent emergent TEVAR primarily due to organ malperfusion (42%) and aortic rupture (33%). Whereas, the US CTAG Dissection IDE trial, which is a multicenter study involving 50 patients from 26 investigative sites in US, reported a higher incidence of malperfusion (82%) [6].

The unstable hemodynamics in complicated BAAD could lead to complicate the determination of the precise SG size. Consequently, a considerable mismatch in aortic diameter between proximal and distal landing sites is not rare. Therefore, it is crucial for the TEVAR device to be readily available and adaptable to its size range. In this context, the CTAG device stands out with its unique 6–33% oversizing range, facilitating an optimal radial fit for treatment of a fragile dissected aorta. Off-the-shelf tapered devices further enhance versatility, accommodating a proximal to distal aortic diameter variance of up to 9.5 mm with a single device, making them well suited for complex cases such as BAAD [8]. Noteworthy, although exact aortic measurements were not revealed due to the nature of PMS research, our registry data reveal that more than half of the patients (55.8%) were able to be treated with a single device and it presented an advantage to cover entry tear immediately in emergency settings. This may have reduced the covered aortic length. In addition, the capability to close entry tear in a single procedure is helpful to prevent RTAD due to entry-mediated false lumen pressure increase before the second deployment of a SG [9].

In terms of perioperative 30-day mortality after TEVAR for complicated BAAD, various researchers have reported a wide range, spanning from 5.5 to 18.0% [6, 10–13]. The US CTAG dissection IDE trial described a 30-day mortality rate of 8%, with fatalities attributed to mesenteric infarction, RTAD with rupture, type Ia endoleak with rupture, and massive pulmonary embolism. Our study similarly demonstrated a high survival rate, with a 30-day mortality of 7.0%, meeting the specified target goal of < 25% as outlined in the literature and Society of Vascular Surgery master file sources [14, 15]. Preoperative risk factors associated with increased mortality after TEVAR have been documented, including a preoperative shock, aortic rupture, mesenteric ischemia, aorta diameter ≥ 5.5 cm, and acute renal failure [12, 13].

There are several serious perioperative complications following TEVAR for complicated BAAD that could result in direct mortality. Foremost among these is RTAD, which is of utmost importance to avoid [9, 16]. Notably, this study recorded no instances of RTAD (0%) during the follow-up periods, suggesting the efficacy of the CTAG device. This device boasts low spring-back force, a wide oversizing range providing optimal radial force, and off-the-shelf tapered devices that enable adjustment of proximal to distal aortic diameter, as mentioned previously [8]. The US CTAG dissection IDE trial reported a 6% incidence of RTAD (3 out of 50 patients), excluding de novo type A dissection. Previous reports have identified several risk factors associated with RTAD, including more proximal landing zones, an ascending diameter exceeding 4 cm, the presence of partial lumen thrombosis, more extensive dissection, and female sex [9, 17]. In terms of SCI, this study identified a low paraplegia rate of 2.4%. This outcome may be attributed to the broad size range of CTAG device and its tapered characteristics, enabling the treatment of complicated BAAD cases with a single device in over half of the instances. A potential explanation for this observed outcome could be the avoidance of excessive treatment length (> 30 cm), as documented as a risk factor for SCI [18]. It is essential to acknowledge, however, that the emergency setting may not provide detailed information concerning the segmental artery, such as the artery of Adamkiewicz [19].

The long-term results revealed favorable rates of true lumen diameter enlargement with a low re-intervention rate. These outcomes may be attributed to a few type I endoleaks through proximal/distal wall apposition and customized oversizing based on patient anatomy, accommodating changes in the true lumen diameters, both characteristic features of the CTAG device [8]. The US CTAG dissection IDE trial demonstrated an average minimum true lumen area enlarged by 206.3 mm^2^ and an average maximum false lumen area reduction of 313.4 mm^2^ from pre-TEVAR to 3 years [6]. In this study, only one patient (2.4%) exhibited false lumen enlargement greater than 5 mm and favorable aortic remodeling was eventually achieved with the false lumen regressing without fatal adverse events, including aortic rupture. Favorable anatomic outcomes are clinically critical, as good remodeling is associated with long-term outcomes, including low re-intervention rates. Berkarda and colleagues have reported that tapering the distal diameter appears to be one factor capable of minimizing the risk of dSINE [20]. From this perspective, the data suggest that the use of the CTAG device can prevent excessive oversizing with tapered SGs and support the reliability of our long-term results. Furthermore, it is widely held that aortic remodeling after TEVAR for complicated BAAD is better in patients with the longer coverage of the downstream aorta [21]. This point was also considered to be an area for further study.

### Limitations

This study has some limitations. Initially, it is important to acknowledge that this is a PMS study with a relatively modest and selective sample size, which was not evenly distributed. Second, this study did not assess the effects of medical treatments, such as anticoagulant, antiplatelet, and antihypertension therapies. Third, the definitions of preoperative comorbidities were not consistent among the facilities. Fourth, this PMS data did not register whether individual malperfusion was improved or not. Fifth, the institutional bias related to the device selection including the length of downstream aorta coverage were not able to be evaluated. Finally, the study did not delve into the variations in treatment approaches and TEVAR procedures across facilities due to insufficient data.

## Conclusions

TEVAR utilizing the CTAG device for complicated BAAD in Japan demonstrated a low incidence of perioperative mortality and complications. Complications directly attributed to the device including RTAD and dSINE were uncommon and the midterm outcomes were deemed satisfactory.

## Supplementary Information

Below is the link to the electronic supplementary material.

Supplementary file1 (DOCX 19 KB)

Supplementary file2 (DOCX 20 KB)

## Data Availability

The data underlying this article cannot be shared publicly because of relevant data protection regulations. However, the data will be shared on reasonable request to the corresponding author with permission from the ethics committee.
