# Correction: Outcomes of thoracic endovascular aortic repair for complicated type B acute aortic dissection from a multicenter Japanese post-market surveillance study

**DOI:** 10.1007/s11748-025-02173-8

**Published:** 2025-06-21

**Authors:** Yoshimasa Seike, Sophie B. Green, Keita Mori, Kimberly Reid, Hitoshi Matsuda, the Vascular Investigators, Hitoshi Matsuda, Naotaka Motoyoshi, Masaki Hata, Hiroyuki Kamiya, Ichiro Ideta, Joji Fukada, Genta Chikazawa, Hiroshi Ishitoya, Takafumi Masai, Tetsuo Sonomura, Kenjiro Kaneko, Yoshikatsu Saiki, Hirokazu Minamimura, Shoichi Takahashi, Masanao Toma, Masafumi Morita, Yutaka Makino, Kazuo Abe, Shinichi Iwakoshi, Toshihiro Funatsu, Keiji Iwata, Ryuta Kiuchi, Kei Kazuno, Yoshiharu Nishimura, Masao Yoshitatsu, Hisashi Satoh, Shinichiro Shimura, Tetsuya Horai

**Affiliations:** 1 Department of Cardiovascular Surgery, National Cerebral and Cardiovascular Center, 6-1 Kishibeshimmachi, Suita, Osaka 564-8565 Japan; 2 W.L. Gore & Associates, Flagstaff, Arizona USA

**Correction: General Thoracic and Cardiovascular Surgery** 10.1007/s11748-025-02123-4

The article ‘Outcomes of thoracic endovascular aortic repair for complicated type B acute aortic dissection from a multicenter Japanese post-market surveillance study’, written by Yoshimasa Seike, Sophie B. Green, Keita Mori, Kimberly Reid, Hitoshi Matsuda on behalf of the Vascular Investigators, was originally published under exclusive license to The Japanese Association for Thoracic Surgery. As a result of the subsequent decision to publish the article under the open access model, the article’s copyright notice was changed on 02 June 2025 to © The Author(s) 2025 and the article is now distributed under a Creative Commons Attribution 4.0 International License, which permits use, sharing, adaptation, distribution and reproduction in any medium or format, as long as you give appropriate credit to the original author(s) and the source, provide a link to the Creative Commons licence, and indicate if changes were made. The images or other third party material in this article are included in the article’s Creative Commons licence, unless indicated otherwise in a credit line to the material. If material is not included in the article’s Creative Commons licence and your intended use is not permitted by statutory regulation or exceeds the permitted use, you will need to obtain permission directly from the copyright holder. To view a copy of this licence, visit http://creativecommons.org/licenses/by/4.0/. The original article has been corrected.

