# Supplementary material for: Outcomes of thoracic endovascular aortic repair for complicated type B acute aortic dissection from a multicenter Japanese post-market surveillance study
Source: Gen Thorac Cardiovasc Surg. 2025 Feb 1;73(8):592–600. doi: 10.1007/s11748-025-02123-4 (PMC12289800; doi:10.1007/s11748-025-02123-4)
Supplement: Supplementary file 1 — Supplementary file1 (DOCX 19 KB) [file 11748_2025_2123_MOESM1_ESM.docx]

**Table S1: Landing zones for deployed stent graphs**

|  | Proximal Landing Zone  N=42 ^*^ | Distal Landing Zone  N=42 ^*^ |
| --- | --- | --- |
| Zone 0 | 1 (2.4%) | 0 (0%) |
| Zone 1 | 0 (0%) | 0 (0%) |
| Zone 2 | 20 (47.6%) | 0 (0%) |
| Zone 3 | 20 (47.6%) | 0 (0%) |
| Zone 4 | 1 (2.4%) | 1 (2.4%) |
| Zone 5 | 0 (0%) | 5 (11.9%) |
| Zone 6 | 0 (0%) | 2 (4.8%) |
| Zone 7 | 0 (0%) | 5 (11.9%) |
| Zone 8 | 0 (0%) | 7 (16.7%) |
| Zone 9 | 0 (0%) | 6 (14.3%) |
| Zone 10 | 0 (0%) | 10 (23.8%) |
| Zone 11 | 0 (0%) | 3 (7.1%) |
| Zone 12 | 0 (0%) | 3 (7.1%) |

^*^ Data not reported for 1 subject who did not survive the index procedure.
