# Supplementary material for: Outcomes of thoracic endovascular aortic repair for complicated type B acute aortic dissection from a multicenter Japanese post-market surveillance study
Source: Gen Thorac Cardiovasc Surg. 2025 Feb 1;73(8):592–600. doi: 10.1007/s11748-025-02123-4 (PMC12289800; doi:10.1007/s11748-025-02123-4)
Supplement: Supplementary file 2 — Supplementary file2 (DOCX 20 KB) [file 11748_2025_2123_MOESM2_ESM.docx]

Appendix Table S2: Principal Investigators lists and Affiliations

| Investigator Name | Division/Department | Facility |
| --- | --- | --- |
| Naotaka Motoyoshi, M.D., Ph.D. | Department of Cardiovascular Surgery | Osaki Citizen Hospital |
| Masaki Hata, M.D. | Department of Cardiovascular Surgery | Sendai Kousei Hospital |
| Hiroyuki Kamiya, M.D., Ph.D. | Department of Cardiac Surgery | Asahikawa Medical University |
| Ichiro Ideta, M.D. | Division of Cardiovascular Surgery | Saiseikai Kumamoto Hospital |
| Joji Fukada, M.D., Ph.D. | Department of Cardiovascular Surgery | Otaru City General Hospital |
| Genta Chikazawa, M.D., Ph.D. | Department of Cardiovascular Surgery | The Sakakibara Heart Institute of Okayama |
| Hiroshi Ishitoya, M.D., Ph.D. | Department of Cardiovascular Surgery | Ehime Prefectural Central Hospital |
| Takafumi Masai, M.D., Ph.D. | Department of Cardiovascular Surgery | Osaka Police Hospital |
| Tetsuo Sonomura, M.D., Ph.D. | Department of Radiology | Wakayama Medical University |
| Kenjiro Kaneko, M.D. | Vascular Surgery | Shin-yurigaoka General Hospital |
| Yoshikatsu Saiki, M.D., Ph.D. | Division of Cardiovascular Surgery | Graduate School of Medicine, Tohoku University |
| Hirokazu Minamimura, M.D., Ph.D. | Department of Cardiovascular Surgery | Bell Land General Hospital |
| Shoichi Takahashi, M.D., Ph.D. | Department of Cardiovascular Surgery | Hoshi General Hospital |
| Masanao Toma, M.D. | Department of Cardiology | Hyogo Prefectural Amagasaki General Medical Center |
| Masafumi Morita, M.D., Ph.D.* | Department of Surgery, Cardiovascular Center | Kyoto Katsura Hospital |
| Yutaka Makino, M.D., Ph.D. | Department of Cardiovascular Surgery | Oji General Hospital |
| Hitoshi Matsuda, M.D., Ph.D. | Department of Cardiovascular Surgery | National Cerebral and Cardiovascular Center |
| Kazuo Abe, M.D., Ph.D. | Department of Cardiovascular Surgery | Yamagata Prefectural Central Hospital |
| Shinichi Iwakoshi, M.D. | Department of Diagnostic and Interventional Radiology | Nara Medical University |
| Toshihiro Funatsu, M.D., Ph.D. | Department of Cardiovascular Surgery | Rinku General Medical Center |
| Keiji Iwata, M.D., Ph.D. | Department of Cardiovascular Surgery | Sakai City Medical Center |
| Ryuta Kiuchi, M.D., Ph.D.* | Department of Cardiovascular Surgery | NewHeart Watanabe Institute |
| Kei Kazuno, M.D. | Department of Cardiovascular Surgery | IMS Fujimi General Hospital |
| Yoshiharu Nishimura, M.D., Ph.D. | Department of Cardiovascular Surgery | Wakayama Medical University |
| Masao Yoshitatsu, M.D., Ph.D. | Department of Cardiovascular Surgery | National Hospital Organization Osaka National Hospital |
| Hisashi Satoh, M.D., Ph.D. | Department of Cardiovascular Surgery | Higashi Takarazuka Satoh Hospital |
| Shinichiro Shimura, M.D., Ph.D.* | Department of Cardiovascular Surgery | School of Medicine, Tokai University |
| Tetsuya Horai, M.D., Ph.D.* | Department of Cardiovascular Surgery | National Center for Global Health and Medicine |

* No longer affiliated with study
